# Experimental demonstration of accelerated extinction in source-sink metapopulations

**DOI:** 10.1002/ece3.713

**Published:** 2013-08-22

**Authors:** John M Drake, Blaine D Griffen

**Affiliations:** Odum School of Ecology, University of GeorgiaAthens, Georgia, 30602-2202

**Keywords:** *Daphnia magna*, extinction, metapopulation, microcosm, source-sink dynamics

## Abstract

Population extinction is a fundamental ecological process which may be aggravated by the exchange of organisms between productive (source) and unproductive (sink) habitat patches. The extent to which such source-sink exchange affects extinction rates is unknown. We conducted an experiment in which metapopulation effects could be distinguished from source-sink effects in laboratory populations of *Daphnia magna*. Time-to-extinction in this experiment was maximized at intermediate levels of habitat fragmentation, which is consistent with a minority of theoretical models. These results provided a baseline for comparison with experimental treatments designed to detect effects of concentrating resources in source patches. These treatments showed that source-sink configurations increased population variability (the coefficient of variation in abundance) and extinction hazard compared with homogeneous environments. These results suggest that where environments are spatially heterogeneous, accurate assessments of extinction risk will require understanding the exchange of organisms among population sources and sinks. Such heterogeneity may be the norm rather than the exception because of both the intrinsic heterogeneity naturally exhibited by ecosystems and increasing habitat fragmentation by human activity.

## Introduction

Population extinction structures biological communities (Chave 2004; Chase 2007), landscapes (Condit et al. 2002), and the worldwide distribution of biodiversity (Brooks et al. 2006; Davies et al. 2006). Although fundamental to progress in community ecology (Holyoak et al. 2005) and biogeography (Hubbell 2001; Volkov et al. 2003), and critical for informing conservation actions in increasingly fragmented landscapes (Fahrig 2003; International Union for the Conservation of Nature (IUCN) 2006), the theory of population extinction has rarely been tested with controlled experiments (Belovsky et al. 1999; Drake 2005; Griffen and Drake 2008a; Drake and Griffen 2010). Further, most extinction models assume that populations are well-mixed (Dennis et al. 1991; Sabo et al. 2004), though there is now considerable evidence that the persistence of many populations is determined by exchange of individuals among connected populations and a balance between production in source habitats and decline in sink habitats, due to the inevitable spatial distribution of organisms over intrinsically heterogeneous spaces (Pulliam 1996; Harrison and Taylor 1997; Gonzalez and Holt 2002; Hanski and Ovaskainen 2003; Holt et al. 2003; Tittler et al. 2006; Cronin 2007).

Time-to-extinction in subdivided populations typically is predicted to decrease with the degree of subdivision, *ceteris paribus* (reviewed in Ovaskainen 2002a). In nature, population subdivision is often accompanied by habitat loss, confounding empirical attempts to measure the effects of habitat subdivision (Fahrig 2003). In this article, we follow Fahrig (2003) in using “fragmentation” to refer to the subdivision of a population, separate from effects of habitat loss or disruption of resource supplies. Models show that if demographic stochasticity is the primary cause of extinction and patches are unconnected, then increasing fragmentation universally leads to a decrease in the mean extinction time (Quinn and Hastings 1987; Burkey 1999). If patches are connected, however, the picture is more complicated. Under some conditions (e.g., intraspecific competition and distance-weighted migration), persistence declines with the number of patches for a given total patch area (Burkey 1989; Etienne and Heesterbeek 2000; Molofsky and Ferdy 2005), while under other conditions (e.g., Allee effects in within-patch dynamics), time-to-extinction is maximized at an intermediate level of fragmentation (Etienne and Heesterbeek 2000; Ovaskainen 2002a; Zhou and Wang 2005). Previous experiments have shown persistence to be greatest in intact populations compared with fragmented populations of the same size (Forney and Gilpin 1989; Burkey 1997) or have failed to detect an effect of fragmentation (Griffen and Drake 2009).

In contrast, the effect of heterogeneity in patch quality on time-to-extinction has not been tested. A recent development that laid the groundwork for the empirical results reported here shows that dynamics of source-sink systems may in fact be described by one of several standard models, subject to an adjustment that accounts for the effect of spatial heterogeneity (Frank and Wissel 2002; Ovaskainen 2002b; Frank 2005). In this formulation, when heterogeneity is reduced to zero, the source-sink model and the standard homogeneous patch theory are equivalent (Ovaskainen 2002b; Frank 2005). According to this theory, source-sink exchange acts on extinction through its effect on “classical” parameters, such as carrying capacity. Further, standard models universally agree that extinction time increases with carrying capacity, basically because as the carrying capacity gets larger the probability of a stochastic excursion from equilibrium of sufficient magnitude to reach the extinction threshold (typically zero) becomes very small (Tier and Hanson 1981; Foley 1994; Lande et al. 2003). We call this the *mechanism of effective carrying capacity*. In source-sink systems, this phenomenon is more complicated: source-sink theory is indeterminate with respect to the effect of source-sink structure on the carrying capacity of the total metapopulation (Holt 1985). Specifically, whether or not the collective carrying capacity exceeds the sum of the carrying capacities of the habitat patches considered in isolation depends on both absolute dispersal rates and relative rates of local population growth (Holt 1985; Pulliam 1988). From this theory, it follows that to determine the effect of source-sink structure on time-to-extinction requires ascertaining whether or not the source-sink structure increases or decreases carrying capacity. If source-sink structure increases effective carrying capacity (Dias 1996), one predicts the time-to-extinction to increase as a result, whereas a decrease in effective carrying capacity due to source-sink structure should reduce the time-to-extinction. To our knowledge, this prediction had not been tested prior to this study.

Furthermore, there is no reason to restrict attention to effects on carrying capacity. Temporal population variability also affects extinction risk, primarily by increasing the frequency of far-from-equilibrium excursions which place a population in the extinction vicinity. By extension, we therefore suggest that if source-sink structure should increase overall temporal variability, then the frequency at which the metapopulation will visit the small population sizes where it is vulnerable to extinction will reduce time-to-extinction, a prediction consistent with (but not equivalent to) the stochastic occupancy model of Ovaskainen (2002b). Conversely, we suggest that if source-sink structure should decrease metapopulation variability, then time-to-extinction will increase. We call this the *mechanism of effective variability*.

Finally, source-sink systems may vary in the degree of resource concentration, which is separate from whether differences between source and sink patches exist at all. For instance, source patches in source-sink systems might be characterized by many low-resource habitats that each have a moderate abundance of resources, or alternatively, the same quantity of resources may be more highly concentrated into a few sites, giving rise to a few high-resource habitats. Thus, source-sink habitat structure may be best thought of as a continuum, with well-mixed-resource environments at one extreme (i.e., no source-sink dynamics) and strong resource concentration at the other extreme (e.g., all resources in a single-source patch with all other patches representing sinks).

This reasoning leads to three more specific, testable hypotheses:

H1 *Classical fragmentation hypotheses*. Time-to-extinction will decrease with increasing habitat fragmentation because local carrying capacities are reduced (Burkey 1989; Etienne and Heesterbeek 2000; and Molofsky and Ferdy 2005). This hypothesis is not universal and in some special cases theory predicts that time-to-extinction is maximized at an intermediate level of fragmentation, for instance when colonization is spatially correlated (Etienne and Heesterbeek 2000; Ovaskainen 2002a; Zhou and Wang 2005.)
H2 *Source-sink hypotheses*.
(A) *Mechanism of effective carrying capacity*. If average total population size is increased by source-sink structure, time-to-extinction will be greater in source-sink environments than in constant-resource environments, regardless of the degree of fragmentation. However, if total population size is decreased by source-sink structure, time-to-extinction will be greater in constant-resource environments than in source-sink environments, regardless of the degree of fragmentation.
(B) *Mechanism of effective variability*. If total population variability is increased by source-sink structure, time-to-extinction will be less in source-sink environments than in constant-resource environments, regardless of the degree of fragmentation. However, if total population variability is decreased by source-sink structure, time-to-extinction will be greater in constant-resource environments than in source-sink environments, regardless of the degree of fragmentation.
H3 *Resource concentration hypotheses*.
(A) *Mechanism of effective carrying capacity*. If average total population size is increased by source-sink structure, time-to-extinction will increase with resource concentration in a multi-patch environment due to the mechanism of effective carrying capacity. However, if total population size is decreased by source-sink structure, time-to-extinction will decrease with resource concentration in a multipatch environment.
(B) *Mechanism of effective variability*. If total population variability is increased by source-sink structure, time-to-extinction will decrease with resource concentration in a multipatch environment due to the mechanism of effective variability. However, if total population variability is decreased by source-sink structure, time-to-extinction will increase with resource concentration in a multipatch environment due to the mechanism of effective variability.

We conducted an experiment in which populations of a model zooplankton species (*Daphnia magna*) were reared under different levels of fragmentation and resource concentration. In our experiment, observed extinction times in homogeneous, subdivided habitats were maximized at an intermediate level of fragmentation – a pattern consistent with some models, but contrary to most of the existing extinction theory (Hypothesis 1). Observed extinction times in heterogeneous, subdivided habitats were more consistent with standard predictions. Particularly, time-to-extinction declined in source-sink environments compared with homogeneous, subdivided habitats (Hypothesis 2) and declined further along a gradient of resource concentration within heterogeneous, subdivided habitats (Hypothesis 3).

## Material and Methods

### Experimental setup

**Figure 1:**
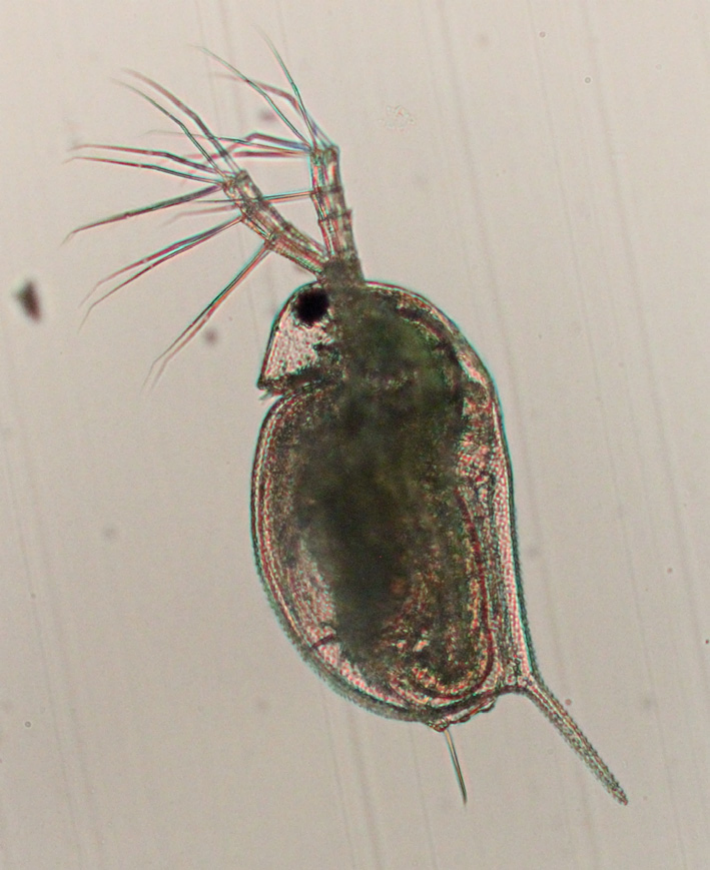
We studied extinction in experimental metapopulations of *Daphnia magna*. (Image: Tad Dallas)

**Figure 2:**
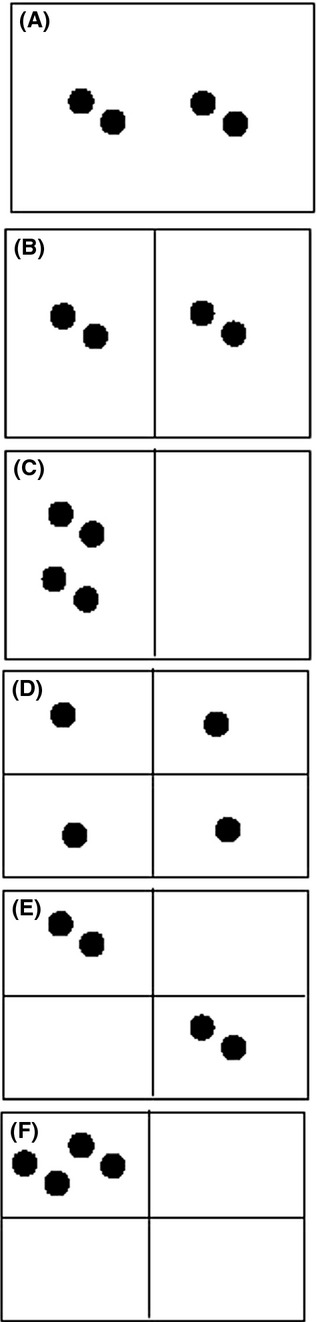
The full experiment comprised six treatments crossing fragmentation (1, 2, or 4 chambers) and number of chambers fed (1, 2, or 4). Each dot represents a daily resource provision of 100 μL of suspended *Spirulina*.

To distinguish metapopulation effects (i.e., population fragmentation due to habitat subdivision), source-sink effects (i.e., spatial asymmetry in resource distribution), and resource concentration effects, we performed an experiment with clonal metapopulations of the parthenogenetic crustacean *D. magna* (Fig. 1) under different resource supply treatments and habitat configurations crossing degree of fragmentation and heterogeneity in patch quality (Fig. 2). Each of the six treatment combinations was replicated ten times (*n* = 60). The experimental setup comprised populations of genetically identical animals reared in 700 mL (31.5 × 27.5 × 1 cm) microcosms constructed from clear Plexiglas and subdivided into chambers, depending on treatment. Chambers within microcosms were connected through 4 holes (2 mm diameter). Daily migration between adjacent compartments in these chambers is ∼23% for juveniles and ∼3% migration for small adults (Griffen and Drake 2009). To randomize effects of variation in the laboratory, microcosms were assigned to one of 10 blocks, each of which occupied a designated location on the lab bench. Chambers were stacked horizontally. Both block position and vertical location within the block were randomly assigned. Each microcosm was fed daily 0.8 μg of inactivated blue–green alga (*Spirulina* sp.; 10.15% N, 44.96% C) suspended in 400 μL of deionized water, supplying the populations with adequate nutrition for population growth, but eliminating the confounding effect of endogenous consumer-resource feedbacks. Previous experiments in this system suggested that such low food amounts would facilitate fairly rapid extinction (Griffen and Drake 2008b), and would therefore accentuate the extinction process. Under these conditions, the generation time is approximately 2 weeks (Griffen and Drake 2008b). The total quantity of food was divided among one, two, or four chambers depending on treatment. Because the number of patches was an experimental treatment, we could not control for initial population size at both the patch (subchamber) and chamber levels simultaneously. As subdivided chambers were constructed to function nearly independently, (i.e., migration small enough that coupling is weak), we elected to control for initial population size at the subchamber level, inoculating each subchamber with five individuals regardless of the number of subchambers in a metapopulation. Other work has shown that an initial population size of *N*_0_ = 5 is adequate to remove effects of initial population size in this system (Drake et al. 2011). Thus, populations in all chambers, even those with only a single compartment that therefore had *N*_*0*_ = 5, had initial population sizes that were large enough to overcome transient effects of initial conditions. Weekly censuses were performed for 22 weeks by separately counting the numbers of juveniles and of adults in the population six times with a hand tally counter. An extinction event was scored only when all six counts were 0. Some chambers were contaminated by green algae before extinction occurred. These chambers were immediately removed from the experiment. We also counted the number of gravid adults at each census.

### Statistical analysis

Metapopulation size on each censusing date was scored as the average of the six recorded counts, summed over all chambers. Average metapopulation size was obtained as the time average of these estimates. Metapopulation variability was scored as the coefficient of variation in metapopulation size over time. Effects of experimental treatments on average metapopulation size and variability were tested using linear mixed and fixed effects models (Pinheiro and Bates 2004). Chambers removed from the experiment due to algae contamination were treated as right-censored observations. Each of hypotheses *H*_1_ through *H*_3_ was tested using Cox proportional hazards regression on the applicable subset of populations (Therneau and Grambsch 2001). The standard partial likelihood estimates were obtained using the R function coxph (R Development Core Team). For hypotheses involving more than one treatment, both interactions and main effects were estimated. Habitat fragmentation was alternately scored as the number of chambers (1, 2, or 4) or the reciprocal (1, 0.5, or 0.25), which we think of as “habitat intactness.” The concept of intactness allows the interaction of fragmentation with number of chambers fed to be interpreted as the fraction of habitat comprised of sources. Following Therneau and Grambsch (2001), possible violations of the proportional hazards assumption of the Cox regression were investigated by testing the correlation between the scaled Schoenfeld residuals for each experimental treatment and time. A significant correlation for any variable was interpreted as evidence that the proportional hazards assumption was violated by that variable. Data and R code for reproducing results reported in this article can be downloaded from (http://daphnia.ecology.uga.edu/drakelab/datapage).

## Results

### Population size and variability

**Figure 3:**
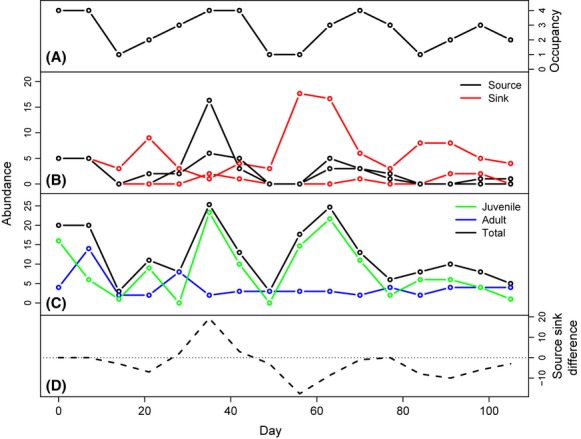
Population dynamics in a representative four-chamber microcosm with two sources and two sinks. (A) Fluctuations in occupied number of chambers showed cycles with ∼5-week period. (B) Fluctuations in abundance in each of four chambers show that population peaks may occur in both sources and sinks. (C) Fluctuations in total metapopulation size were strongly correlated with fluctuations in the abundance of juveniles. (D) The relative abundance in sources versus sinks (calculated by summing the total abundance in sources and subtracting the total abundance in sinks) showed little overall variation in abundance between sources and sinks.

The dynamics and spatial variation in a typical metapopulation are illustrated in Figure 3. These data are from a four-chamber microcosm in which two of the four chambers were fed. The top panel (Fig. 3A) shows the number of chambers that were occupied (*N* > 0) on each sampling date between the start of the experiment (Day 0) and the censoring date of this microcosm (Day 105). Recalling that the generation time under these conditions is about 2 weeks, the occupancy data appear to show multigeneration cycles with a period of approximately two and a half generations. The second panel decomposes this cycle into its subpopulation components (Fig. 3B). This plot shows that the occupancy cycles reflect cycles in abundance overall and are not driven by either the source or sink populations exclusively, as the first peak in abundance occurs in a source and the second and third peaks are primarily due to juveniles trapped or sojourning in a sink. How this occurs is illustrated in the third panel (Fig. 3C), which aggregates abundance over sources and sinks by age-class. This plot shows that the cycles in population abundance are driven by birth cohorts (“baby booms”) occurring around days 21, 35, 56, and 84. Comparing Figure 3B with Figure 3C, one sees that the first, third, and fourth of these cohorts are largely confined to one of the sinks, whereas the second cohort remains in a source. Thus, the peaks in occupancy can occur in either sources or sinks and reflect the population inertia inherent in the aggregate dynamics. We observe that after approximately 5 weeks of transient oscillations, the abundance of adults in this metapopulation remained relatively stable. The net effect of these dynamics on the spatial distribution of individuals between sources and sinks is therefore equivocal (Fig. 3D), although averaged over the entire experiment, abundance in sources was greater than abundance in sink by approximately 2×. The dynamics of total abundance of all individuals in all chambers (black line in Fig. 3C) is dominated neither by source nor sink subpopulations, as illustrated in the difference between the number of individuals in sources and the number of individuals in sinks over time. Further, 10 of 18 gravid individuals observed in this microcosm were found in sink habitats, suggesting that production might occur in both sources and sinks. Similarly, 39 of 60 observations of adults were in sink habitats.

**Figure 4:**
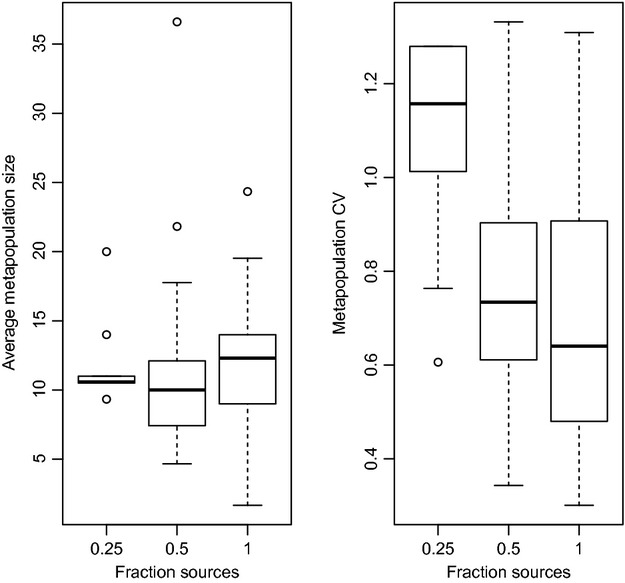
Effect of the fraction of patches that were sources on the average metapopulation size and metapopulation variability in treatments that reveal the effect of source-sink dynamics (treatments D, E, and F).

Data were pooled to test for effects of experimental treatments on average metapopulation size and variability. Effects of experimental treatments on average metapopulation size were first estimated using a mixed-effects model in which experimental treatments and position within a block were treated as fixed and a random intercept was fit for the effects of block. These models failed to detect evidence for any effect of block (likelihood ratio of 1.11 on 1 df; *P* = 0.29) or height on average metapopulation size (Table S1) or variability (Table S2). However, experimental treatments did influence average metapopulation size and variability. Particularly, average metapopulation size significantly declined with intactness (meaning that population size increased with fragmentation), but increased with the fraction of habitat patches that were sources (Table S1; Fig. 4). Metapopulation variability, in contrast, increased with intactness and declined with the fraction of habitat patches that were sources (Table S2; Fig. 4). Three populations went extinct in the first censusing interval. As the variance in these populations could not be calculated, these replicates were dropped from the analysis. As expected, time-to-extinction increased with average metapopulation size and decreased with metapopulation variability with the size of effect for variability ∼1.8× the effect of average metapopulation size (Cox proportional hazards model using the logarithm of average metapopulation size and logarithm of coefficient of metapopulation size as predictors; Table S3). In this model, the proportional hazards assumption was weakly violated for average metapopulation size. Inspection of residuals showed that this effect was small.

### Hypothesis 1: fragmentation

**Figure 5:**
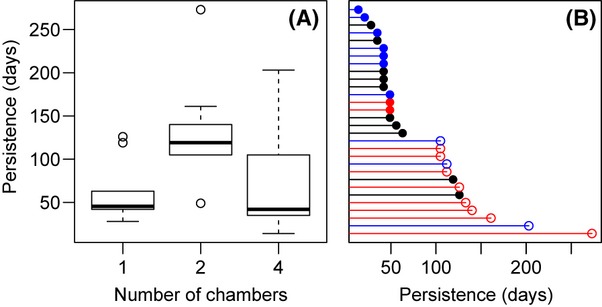
Effect of habitat fragmentation on persistence of experimental populations. (A) Persistence is greatest at all quartiles in populations with an intermediate level of fragmentation. (B) Additionally, populations with two chambers (red) were more likely to be censored (open circles) than populations with one chamber (black) or populations with four chambers (blue), which typically were observed until extinct (filled circles).

Extinction was observed in 19 of 30 (63%) metapopulations in treatments {A, B, D} (chambers with evenly distributed resources increasing in fragmentation). In this and subsequent analyses, nonextinct populations were right-censored (i.e., populations terminated before extinction were appropriately treated in statistical analysis). For this analysis, we treated habitat fragmentation as an unordered factor because analyses treating it as a continuous variable violated the proportional hazards assumption. This analysis showed that time-to-extinction increased in microcosms with two chambers compared with microcosms with one chamber (*P* = 0.005), but not for microcosms with four chambers (*P* = 0.72; Fig. 5A; Table S4). Additionally, microcosms with intermediate levels of fragmentation (two chambers) were also more likely to persist until the experiment was terminated (Fig. 5B). Thus, in small microcosms, the most persistent populations were those with an intermediate level of fragmentation.

### Hypothesis 2: source-sink

Extinction was observed in 25 of 40 (63%) metapopulations in treatments {B, C, D, E} (multipatch chambers contrasting evenly distributed and concentrated resources). There was no evidence for a main effect of intactness on extinction (*P* = 0.073), though the time to extinction decreased as the number of resource patches increased from two to four (*P* = 0.035; Table S5). Time-to-extinction also increased with the interaction between intactness and number of resource patches, which is the fraction of habitat comprised of sources (*P* = 0.010; Table S5). Thus, source-sink metapopulations went extinct faster than constant resource metapopulations. Residual analysis provided no reason to reject the assumption of proportional hazards for either effect or the interaction term (*P*_intactness_ = 0.94, *P*_number of sources_ = 0.71, *P*_fraction of habitat sources_ = 0.40). 

### Hypothesis 3: resource concentration

Extinction was observed in 24 of 30 (80%) metapopulations in treatments {D, E, F} (chambers with a consistent level of fragmentation, increasing in resource concentration). As above, we treated fraction of habitat comprised of sources as a categorical variable (Table S6). Time-to-extinction in the most resource-concentrated treatment (1 of 4 or 25% of patches a source) was significantly shorter than in either of the other two treatments, and time-to-extinction in these two treatments (50% and 100% sources) were not different from each other. That is, the most severely asymmetrical source-sink metapopulations went extinct faster than metapopulations with mildly asymmetrical resource distributions and metapopulations with homogeneous resource environments. Together with the results in Figure 4, this suggests that effects of experimental treatments more likely were mediated by metapopulation variability than by metapopulation size. Indeed, a further analysis of variance, in which the test was restricted to populations in treatments {D, E, F} failed to detect any effect of resource concentration on average population size (*F* = 0.774, *P* = 0.47), but showed a strong effect on the coefficient of variation in population size (*F* = 9.028, *P* = 0.001).

## Discussion

The standard stochastic theory predicts that time-to-extinction in closed, well-mixed populations will be positively correlated with carrying capacity (Tier and Hanson 1981; Lande et al. 2003) and negatively correlated with demographic and environmental variance (Alvarez 2001). The extension to source-sink metapopulations is not straightforward and is an area of ongoing research (Frank and Wissel 2002; Hanski and Ovaskainen 2003; Frank 2005). Classical source-sink models concern only the effect of source-sink structure on carrying capacity, which was shown to be context specific (Holt 1985; Pulliam 1988). Subsequently, Harrison and Taylor (1997) extended this line of thought to speculate about the effects of population variability: “If local populations fluctuate fairly independently of one another, but exchange low to moderate numbers of immigrants, metapopulation structure may have an important stabilizing effect at the regional level even without population turnover. We know of no good examples of this possibility” (p. 35). Analyses reported here show that source-sink structure can indeed act on extinction through its effects on the magnitude of fluctuations.

Experimental data reported here provide some evidence that could guide further theoretical work along these lines. First, we detected an effect of source-sink structure (fraction of habitat comprised of sources) on average population size (Table S1). To our knowledge, this is the first empirical example of this phenomenon. Perhaps more importantly, however, our data show a strong relationship between extinction time and the coefficient of variation in metapopulation size (Table S3). This points to a causal pathway whereby environmental heterogeneity increases temporal metapopulation variability compared with populations in homogeneous environments, which increases vulnerability to extinction. The importance of source-sink structure on effective variability vis-a-vis effective carrying capacity may be quantified by comparing the coefficients of determination for the model of average metapopulation size (*R*^2^ = 0.16; Table S1) and the coefficient of variation in metapopulation size (*R*^2^ = 0.25; Table S2), an improvement in predictability of >50%.

Our analysis of *H*_1_ provides the most intriguing finding: population persistence was maximized at intermediate levels of habitat fragmentation in small microcosms. This finding is consistent with some models (Etienne and Heesterbeek 2000; Ovaskainen 2002a; Zhou and Wang 2005), but to our knowledge this is the first study reporting empirical data confirming such a relationship. Our study, which controls for confounding effects of total available habitat, is therefore a counterexample to the finding by Harrison and Taylor (1997) that metapopulation persistence increases with the number of patches. One potential explanation is that extinction risk was diminished in the two-chamber microcosm relative to the one-chamber microcosm because the spatial separation broke up synchronous overcompensatory fluctuations, and relative to the four-chamber microcosm because the average habitat size of the latter depressed subpopulation size to such a level that local extinction became frequent and habitat patches were commonly empty. The generality of this intermediate fragmentation effect cannot be adequately assessed until additional experiments are performed in other systems. Because conservation strategies must often deal with severe habitat fragmentation, demonstration of this phenomenon in experimental natural systems would be of particular interest.

*Daphnia* have often been studied to understand ecological physiology and the factors that influence growth, survival, and reproduction. This previous work addresses two points that may be pertinent to the results reported here. First, crowding is an important factor in *Daphnia* population dynamics, reducing individual growth and reproduction (Burns 1995, 2000; Martínez-Jerónimo et al. 2000; Preuss et al. 2009). However, the population densities observed in our experiments were much lower than those that induce crowding effects in this species and therefore probably was not a factor leading to extinction in our experiment. Second, phosphorous is often a limiting factor for *Daphnia* growth (Boersma 2000), and nutritional deficits could plausibly have affected extinction in our experiment. If this occurred, the causal chain of events is not clear, as *Daphnia* populations housed by us under similar conditions, but at higher food levels and without migration between subpopulations, have persisted for greater than a year (Griffen and Drake 2008b). More parsimoniously, we submit that extinction in our experimental populations resulted from low reproduction due to both low food availability and nutrient/mineral limitations, combined with fluctuations caused by natural variation in growth and survival (i.e., demographic stochasticity) that become increasingly important as population size decreased (Desharnais et al. 2006).

In conclusion, the findings of this study include the following. First, environmental heterogeneity induced by source-sink population structure decreased average metapopulation size and increased the coefficient of variation in metapopulation size compared with populations in which resources were evenly distributed among habitat patches. Second, we documented highest extinction risk at an intermediate level of habitat fragmentation, the generality of which awaits additional research. Third, the effect of resource concentration on extinction was substantial (Table S6). These results show that classical metapopulation attributes – fragmentation/intactness and habitat size – do indeed affect persistence through their action on metapopulation size and variability. Given the ubiquity of source-sink dynamics in nature and the propensity of source-sink environments to manifest as ecological traps (Schlaepfer et al. 2002), this finding suggests that serious consideration of the configuration of resource supply to populations of conservation concern would be prudent.

## Supporting Information

**Table S1.** Linear model for effects of experimental treatments on average metapopulation size (*n* = 60).

**Table S2.** Linear model for effects of experimental treatments on average metapopulation variability (*n* = 57).

**Table S3.** Cox proportional hazard regression results for effects of average metapopulation size and variability on extinction time (*n* = 57).

**Table S4.** Cox proportional hazard regression results for Hypothesis 1 (small microcosms, *n* = 30). Treatments included in this model are chamber configurations {A, B, D}.

**Table S5.** Cox proportional hazard regression results for Hypothesis 2 (*n* = 40). Treatments included in this model are chamber configurations {B, C, D, E}.

**Table S6.** Cox proportional hazard regression results for Hypothesis 3 (*n* = 30). Treatments included in this model are chamber configurations {D, E, F}.
