# Supplementary material for: Experimental demonstration of accelerated extinction in source-sink metapopulations
Source: Ecol Evol. 2013 Aug 22;3(10):3369–78. doi: 10.1002/ece3.713 (PMC3797484; doi:10.1002/ece3.713)
Supplement: Supplementary file 1 [file ece30003-3369-SD1.pdf]

EXPERIMENTAL DEMONSTRATION OF ACCELERATED EXTINCTION IN SOURCE-SINK METAPOPULATIONS

Online Appendix

John M. Drake[1*] and Blaine D. Griffen[1†]

[1] Odum School of Ecology, University of Georgia, Athens, GA 30602-2202, USA

[*] Author for correspondence: jdrake@uga.edu

[†] Present address: Department of Biological Sciences, University of South Carolina, Columbia, SC, 29208, USA (email: bgriffen@biol.sc.edu)

Table S1. Linear model for effects of experimental treatments on average metapopulation size (*n*=60).

Variable	Coefficient	Std. Err.	*t*	*p*
Number of resource patches	-0.30	0.16	-1.84	0.07
Intactness	-1.57	0.66	-2.3	0.02
Height	-0.04	0.02	-1.85	0.07
Intactness × Number of resource patches = Fraction sources	1.20	0.59	2.04	0.046

R^2: 0.16
F-statistic: 2.672 on 4 and 55 d.f., *p*=0.0415

Table S2. Linear model for effects of experimental treatments on average metapopulation variability (*n*=57)

Variable	Coefficient	Std. Err.	*t*	*p*
Number of resource patches	0.15	0.10	1.41	0.14
Intactness	0.88	0.41	2.13	0.04
Height	-0.02	0.01	-1.21	0.23
Intactness × Number of resource patches = Fraction sources	-1.03	0.37	-2.77	0.008

R^2: 0.25
F-statistic: 4.353 on 4 and 52 d.f., *p*=0.004

Table S3. Cox proportional hazard regression results for effects of average metapopulation size and variability on extinction time (*n*=57).

Variable	Coefficient	Std. Err.	*z*	*p*
log (average metapopulation size)	-0.68	0.25	-2.696	0.007
log (metapopulation cv)	1.26	0.46	2.724	0.006

R^2: 0.26
Likelihood ratio test: 17.15 on 2 d.f., *p*<0.001

Table S4. Cox proportional hazard regression results for Hypothesis 1 (small microcosms, *n*=30). Treatments included in this model are chamber configurations {A, B, D}.

Variable	Coefficient	Std. Err.	*z*	*p*
Chambers=2	-2.18	0.78	-2.795	0.0052
Chambers=4	-0.18	0.49	-0.355	0.72

R^2: 0.35
Likelihood ratio test: 12.7 on 2 d.f., p<0.0018

Table S5. Cox proportional hazard regression results for Hypothesis 2 (*n*=40). Treatments included in this model are chamber configurations {B, C, D, E}.

Variable	Coefficient	Std. Err.	*z*	*p*
Intactness	9.28	5.17	1.80	0.073
Number of resource patches	2.01	0.95	2.11	0.035
Intactness × Number of resource patches = Fraction sources	-8.54	3.32	-2.57	0.010

R^2: 0.28
Likelihood ratio test: 13.1 on 3 d.f., p=0.005

Table S6. Cox proportional hazard regression results for Hypothesis 3 (*n*=30). Treatments included in this model are chamber configurations {D, E, F}.

Variable	Coefficient	Std. Err.	*z*	*p*
Fraction sources=0.5	-1.96	0.59	-3.34	0.0008
Fraction sources=1.0	-2.27	0.61	-3.72	0.0002

R^2: 0.41
Likelihood ratio test: 15.9 on 2 d.f., p=0.0004
